# Genotype and Cardiac Rhabdomyoma Phenotype Analyses in Tuberous Sclerosis Complex Diseases

**DOI:** 10.1155/ijog/2204583

**Published:** 2026-07-01

**Authors:** Xiang Chen, Ruen Yao, Wangtao Sheng, Jiaqi Li, Yiwei Chen, Lijun Fu, Fei Bei

**Affiliations:** ^1^ Department of Neonatology, Shanghai Children′s Medical Center, Shanghai Jiao Tong University School of Medicine, Shanghai, China, shsmu.edu.cn; ^2^ Department of Medical Genetic and Molecular Diagnosis, Shanghai Children′s Medical Center, Shanghai Jiao Tong University School of Medicine, Shanghai, China, shsmu.edu.cn; ^3^ Department of Neonatology, Children′s Hospital of Fudan University, Shanghai, China; ^4^ Department of Cardiology, Shanghai Children′s Medical Center, Shanghai Jiao Tong University School of Medicine, Shanghai, China, shsmu.edu.cn

## Abstract

**Background:**

Cardiac rhabdomyomas (CRs) are one of the typical phenotypes of tuberous sclerosis complex (TSC) diseases. Patients with CR could present various phenotypes and different severities. *TSC1* and *TSC2* are candidate genes for TSC. The genotype and phenotype relationship of the CR phenotype in TSC is unknown.

**Methods:**

*TSC1* and *TSC2* pathogenic and likely pathogenic variants from the HGMD, ClinVar, and LOVD databases were identified (last date: 2024.12.01). After critical exclusion criteria and pathogenicity reanalyses, statistical analyses were performed for enrichment evaluation.

**Results:**

In this study, 1250 variants of *TSC1* were finally included, and 26 variants (20.8%) were reported to cause the CR phenotype. In Exon 15, 5.1% of them were CR‐related (*p* < 0.001), and 7.1% of them were enriched in Exon 18 (*p* = 0.008). After adjusting by the Benjamini–Hochberg FDR method, the FDR‐adjusted *p* values were still significant for Exon 15 (*p* = 0.006) and Exon 18 (*p* = 0.028). Considering the size of each exon, there is no significant enrichment by Poisson model analysis. We included 2690 variants of *TSC2*; 117 variants (4.3%) were reported to cause the CR phenotype. In Exon 41, 12% of variants were CR‐related (*p* = 0.003). The FDR‐adjusted *p* value was not significant for Exon 41 (*p* = 0.11). Considering the size of each exon, in Poisson model analysis, there are significant enrichments detected in Exon 37 (IRR = 2.72 [1.42, 5.19], *p* = 0.003), Exon 38 (IRR = 3.26 [1.65, 6.44], *p* = 0.001), and Exon 41 (IRR = 5.79 [3.11, 10.77], *p* < 0.001). After adjusting by the Benjamini–Hochberg FDR method, significant differences remained in these three exons.

**Conclusion:**

CR phenotypes demonstrated partial enrichment in specific exons, highlighting the importance of exon‐level interpretation during genetic counseling.

## 1. Introduction

Cardiac rhabdomyomas (CRs) are a kind of benign tumor and were first reported in utero in 1982 [1, 2]. It is the most common type of cardiac tumor in infants and usually occurs in the first year of life [3]. It could be diagnosed as early as 19 weeks of gestational age by ultrasonography [4]. After birth, patients with CR can exhibit different phenotypes with varying severity. While most patients were asymptomatic, a minority may present heart failure, cerebral embolization, arrhythmias, and sudden cardiac death due to hemodynamically significant obstructions [5, 6]. The severity of the phenotype is related to the location and size of tumors. If patients successfully survived in early life, this tumor had an approximately 18%~60% rate of regression [7, 8].

CR is often associated with tuberous sclerosis complex (TSC) [9]. Pathogenic or likely pathogenic (P/LP) *TSC1* or *TSC2* gene variants can cause TSC. Patients present skin lesions, seizures, neurodevelopmental delay, and tumor growth in the heart, kidney, and lung. Because CR can be detected in fetuses, it serves as the earliest sign of TSC and alerts to the necessity of ordering genetic tests. Surgery and antiarrhythmic drugs were traditional therapies for symptomatic CR patients. As the TSC1–TSC2 complex is the negative regulator of the mTOR (mammalian target of rapamycin) signaling pathway, the mTOR inhibitor was reported to effectively reduce the tumor size [10].

About 70%–90% of patients with CR have TSC. However, only 50% of patients with TSC had the CR phenotype [3]. Except for *TSC1*/*TSC2*, no variant in other genes was identified to cause CR. Some patients present with both CR and typical TSC features yet receive negative genetic test results [11]. The characteristics of the TSC1/TSC2 genotype and CR phenotype correlations have not been well illustrated [12]. Recent studies focused on how to increase the diagnosis rate of the CR in early pregnancy by ultrasound and the treatment strategy by inhibiting the mTOR signaling pathway. However, few researches explore the relationship between the genotype and phenotype. Therefore, based on public databases and internal data, this study included P/LP variants of *TSC1* and *TSC2* and reviewed their CR phenotype to explore the genotype–CR relationship.

## 2. Methods

### 2.1. Review of Variants

Variants of the *TSC1* and *TSC2* genes were included from the HGMD database (https://www.hgmd.cf.ac.uk/ac/index.php), ClinVar database (https://www.ncbi.nlm.nih.gov/clinvar/), and LOVD database (https://www.lovd.nl/) (2024.11.30). All variants underwent nomination according to the standards of the Human Genome Variation Society (HGVS). The Ensembl number of TSC1 is ENST00000490179.4, and that of TSC2 is ENST00000219476.9. The pathogenicity classification of variants was reanalyzed according to guidelines of the American College of Medical Genetics [13]. The exclusion of criteria includes those variants that cannot map to a certain exon or variants that have uncertain significance. So, in this study, variants matching the following criteria were excluded: (1) variants of unknown significance and benign variants; (2) copy number variants (> 1000 bp); (3) intron variants (non ±1/2); (4) for variants reported in multiple databases, only a single entry was retained; and (5) deletions involving more than one exon.

All variants have been researched by PubMed and ClinVar. We provide the search strategy of one variant in PubMed as an example: “TSC1”[All Fields] AND “c.1525C>T”[All Fields] AND (“cardiac rhabdomyoma”[All Fields] OR “heart tumor”[All Fields] OR “cardiac tumor”[All Fields]). Based on this formula, we searched the CR phenotype of each variant in this study. The searching keywords in ClinVar are “cardiac rhabdomyomas,” “heart tumor,” and “cardiac tumor” (Supporting Information 1: Table S1). By comprehensively applying the above strategies, all site information associated with the CR phenotype can be obtained. Two researchers independently performed the literature mining. Duplicate cases reported across different studies will be removed.

**Figure 1:**
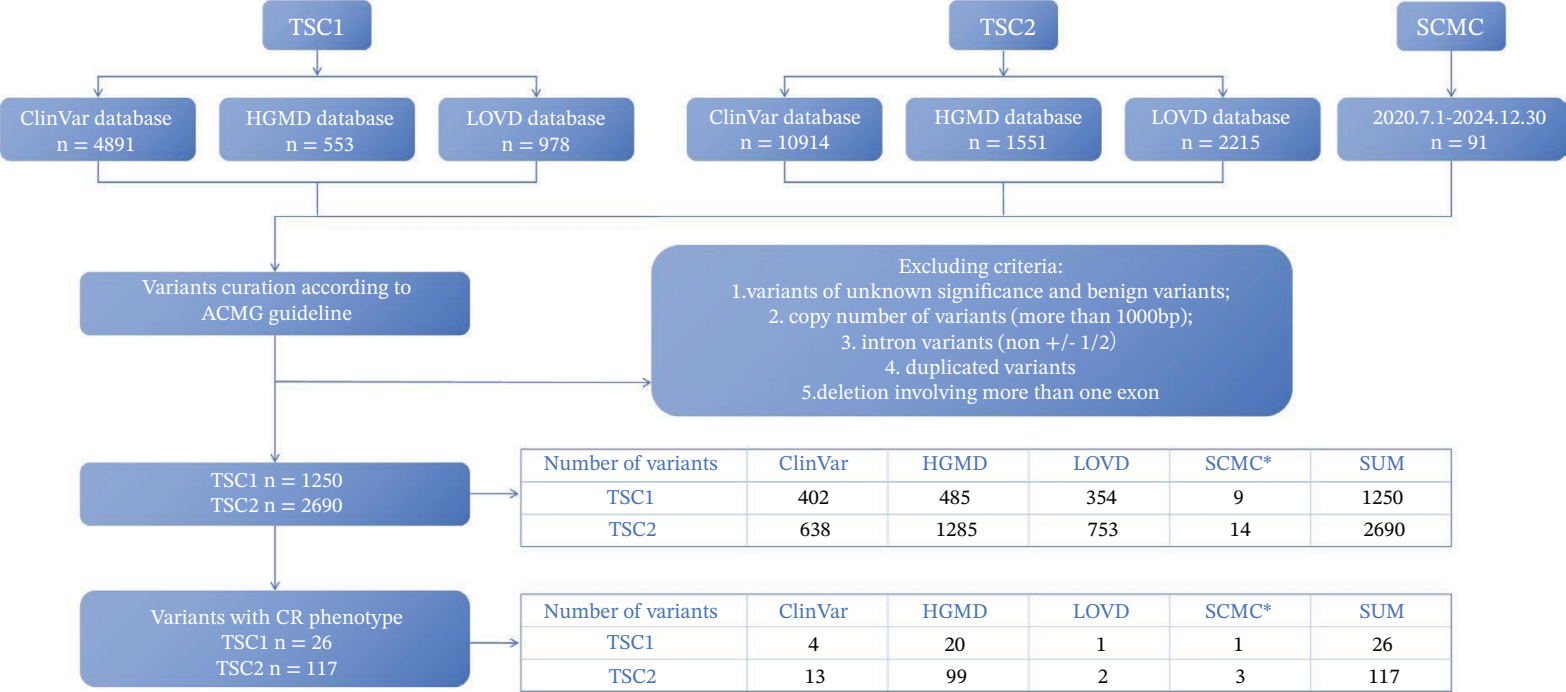
Workflow of candidate variants.  ^∗^SCMC: for those variants reported in public databases were belonging to the public database.

We reviewed genetic data in Shanghai Children′s Medical Center (SCMC) from 2020.07.01 to 2024.12.30. This center underwent both prenatal and postnatal genetic tests. Those patients who underwent genetic tests were suspected of having inherited diseases. This study is a secondary analysis of their genetic data. We recruited all cases with positive genetic findings in the *TSC1* and *TSC2* genes (Figure 1). Clinical information was from electronic medical records. This study has been approved by the ethics committee of SCMC (SCMCIRB‐K2020060‐1). Pretest counseling was performed by clinical physicians. Informed consent was obtained from the patient′s parents.

### 2.2. Genetic Test

The SCMC genetic data were the results of a whole‐exon sequencing (WES) test. The brief standard procedure of WES in our center extracted 3 *μ*g of DNA into fragments of 150–200 bps in size. An adaptor‐ligated paired‐end library was constructed using an Agilent library preparation kit (Agilent Technologies, Santa Clara, CA, United States). Cluster generation was performed on the Illumina cBot system, followed by sequencing on the Illumina HiSeq 2500 platform (Illumina, San Diego, CA, United States). Reads were aligned to the human reference genome (NCBI build 37, hg 19) using BWA, and data quality was evaluated with FastQC. Variant detection and interpretation were conducted using the Ingenuity Variant Analysis platform.

### 2.3. Statistical Analysis

The statistical analyses of the CR phenotype–related variant rates in each exon have been calculated by the chi‐square test or Fisher′s exact test. *p* values were adjusted for multiple comparisons using the Benjamini–Hochberg false discovery rate (FDR) method. Poisson′s regression models were used to assess exon‐level enrichment. Exon‐specific variant counts were the outcome, and the exon length (kilobases) was included as an offset term to account for exon size. Each exon was compared with all remaining exons using a binary indicator variable. Incidence rate ratios (IRRs) were calculated, and *p* values were adjusted for multiple comparisons using the Benjamini–Hochberg FDR method. All statistical analyses were done with R (Version 4.0.3).

## 3. Results

**Figure 2:**
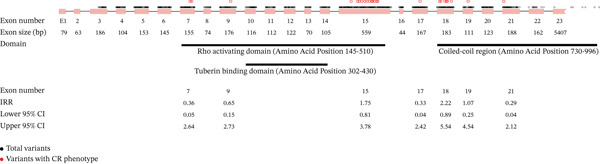
Schematic plot of the cardiac rhabdomyoma (CR)–related and all variants in each exon of *TSC1*. Black circles were all variants of *TSC1* included in this study. Red circles were CR‐related variants in *TSC1*. The exon size, location of domain, and the enrichment of CR‐related variants in each exon are shown below the schematic diagram of the *TSC1* gene.

In this study, we finally included 1250 variants of *TSC1*, 402 from the ClinVar database, 485 from the HGMD database, 354 from the LOVD database, and nine from SCMC (Supporting Information 1: Table S1). A small proportion of variants were reported in more than one database. As for mutation type, there are 827 frameshift variants, 227 stop‐gained, 140 splicing variants, 51 missense variants, and five in‐frame variants. After phenotype screening, 26 variants were reported to cause the CR phenotype (Supporting Information 2: Table S2). Most CR‐related variants were in Exon 15 (50.0%) and Exon 18 (23.1%). Fisher′s test presented the enrichment of CR‐related variants in Exon 15 (*p* < 0.001) and Exon 18 (*p* = 0.008) (Table 1). After adjusting by the Benjamini–Hochberg FDR method, the FDR‐adjusted *p* values were still significant for Exon 15 (*p* = 0.006) and Exon 18 (*p* = 0.028). Considering the size of each exon, there is no significant enrichment by Poisson model analysis (Table 1). However, the IRR value of Exon 15 (IRR = 1.75 [0.81, 3.78]) and Exon 18 (IRR = 2.22 [0.89, 5.54]) were larger than 1. This may suggest a trend toward enrichment of CR‐related variants in Exon 15 and Exon 18. The rest of the CR‐related variants were detected in Exons 7/9/17/19/21. The rate ranged from 3.8% to 7.7%, and no significant enrichment has been identified (Table 1 and Figure 2).

**Table 1:** Exon‐level variant enrichment analysis of the *TSC1* gene using exon length‐adjusted Poisson regression.

Exon	Number of non‐CR‐related variants in each exon	Number of CR‐related variants in each exon	P1 value^a^	P2 FDR‐adjusted values^b^	IRR^c^ (95% CI)	P3 value^d^	P4 FDR‐adjusted values^e^
3	22	0	/	/	/	/	/
4	52	0	/	/	/	/	/
5	68	0	/	/	/	/	/
6	55	0	/	/	/	/	/
7	89	1 (1.1%, 1/89)	1	1	0.36 (0.05, 2.64)	0.313	0.438
8	30	0	/	/	/	/	/
9	81	2 (2.5%, 2/81)	0.684	1	0.65 (0.15, 2.73)	0.684	0.643
10	54	0	/	/	/	/	/
11	34	0	/	/	/	/	/
12	30	0	/	/	/	/	/
13	22	0	/	/	/	/	/
14	32	0	/	/	/	/	/
15	254	13 (5.1%, 13/254)	< 0.001	0.006	1.75 (0.81, 3.78)	0.152	0.438
16	34	0	/	/	/	/	/
17	108	1 (0.9%, 1/108)	0.719	1	0.33 (0.04, 2.42)	0.275	0.438
18	85	6 (7.1%, 6/85)	0.008	0.028	2.22 (0.89, 5.54)	0.086	0.438
19	44	2 (4.5%, 2/44)	0.240	0.560	1.07 (0.25, 4.54)	0.925	0.925
20	62	0	/	/	/	/	/
21	79	1 (1.3%, 1/79)	1	1	0.29 (0.04, 2.12)	0.221	0.438
22	12	0	/	/	/	/	/
23	3	0	/	/	/	/	/
SUM	1250	26	/	/	/	/	/

^a^P1 value—Fisher′s test.

^b^P2 FDR‐adjusted values—*p* values were adjusted for multiple comparisons using the Benjamini–Hochberg false discovery rate (FDR) method.

^c^IRR—incidence rate ratio from Poisson′s regression.

^d^P3 value—Fisher′s test‐unadjusted *p* value derived from Poisson′s regression.

^e^P4 FDR‐adjusted values—*p* value adjusted for multiple comparisons using the Benjamini–Hochberg false discovery rate (FDR) method based on Poisson′s regression.

**Figure 3:**
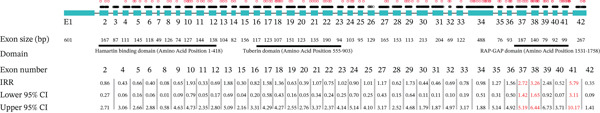
Schematic plot of the cardiac rhabdomyoma (CR)–related and all variants in each exon of *TSC2*. Black circles were all variants of *TSC2* included in this study. Red circles were CR‐related variants in *TSC2*. The exon size, location of domain, and the enrichment of CR‐related variants in each exon are shown below the schematic diagram of the *TSC2* gene.

We included 2690 variants of *TSC2*, 1285 from the HGMD database, 638 from the ClinVar database, 753 from the LOVD database, and 14 from SCMC (Supporting Information 1: Table S1). There are 1514 frameshift variants, 355 stop‐gained, 430 splicing variants, 337 missense variants, and 54 in‐frame variants. We found that 117 variants were reported to cause the CR phenotype (Supporting Information 2: Table S2). These variants are located in every exon of TSC2 except Exons 3/7/14/23/26. In Exon 41, 12% of variants were CR‐related (*p* = 0.003). After adjusting by the Benjamini–Hochberg FDR method, the FDR‐adjusted *p* value was not significant for Exon 41 (*p* = 0.11) (Table 2). Considering the size of each exon, in the Poisson model analysis, there are significant enrichments detected in Exon 37 (IRR = 2.72 [1.42, 5.19], *p* = 0.003), Exon 38 (IRR = 3.26 [1.65, 6.44], *p* = 0.001), and Exon 41 (IRR = 5.79 [3.11, 10.77], *p* < 0.001). After adjusting by the Benjamini–Hochberg FDR method, significant differences remained in these three exons (Table 2). For the rest of the exons, the CR‐related variants account for 0.9%–8.5% in each exon. No significant differences were identified (Table 2 and Figure 3).

**Table 2:** Exon‐level variant enrichment analysis of the *TSC2* gene using exon length‐adjusted Poisson regression.

Exon	Number of non‐CR‐related variants in each exon	Number of CR‐related variants in each exon	P1 value^a^	P2 FDR‐adjusted values^b^	IRR^c^ (95% CI)	P3 value^d^	P4 FDR‐adjusted values^e^
1	1	0	/	/	/	/	/
2	61	3 (4.9%, 3/61)	0.748	0.975	0.86 (0.27, 2.71)	0.796	0.893
3	32	0	/	/	/	/	/
4	47	1 (2.1%, 1/47)	0.721	0.975	0.43 (0.06, 3.06)	0.398	0.819
5	59	2 (3.4%, 2/59)	1	1	0.66 (0.16, 2.66)	0.556	0.892
6	54	1 (1.9%, 1/54)	0.728	0.975	0.40 (0.06, 2.88)	0.364	0.798
7	28	0	/	/	/	/	/
8	65	1 (1.5%, 1/65)	0.525	0.975	0.08 (0.01, 0.58)	0.012	0.114
9	34	1 (2.9%, 1/34)	1	1	0.65 (0.09, 4.63)	0.665	0.893
10	65	5 (7.7%, 5/65)	0.214	0.975	1.93 (0.79, 4.73)	0.150	0.749
11	66	1 (1.5%, 1/66)	0.527	0.975	0.33 (0.05, 2.35)	0.267	0.749
12	58	2 (3.4%, 2/58)	1	1	0.69 (0.17, 2.80)	0.605	0.892
13	50	4 (8%, 4/50)	0.284	0.975	1.88 (0.69, 5.09)	0.215	0.749
14	43	0	/	/	/	/	/
15	75	1 (1.3%, 1/75)	0.374	0.975	0.30 (0.04, 2.16)	0.233	0.749
16	53	2 (3.8%, 2/53)	1	1	0.82 (0.20, 3.31)	0.779	0.893
17	68	4 (5.9%, 4/68)	0.542	0.975	1.58 (0.58, 4.29)	0.367	0.798
18	63	3 (3.2%, 2/63)	0.754	0.975	1.36 (0.43, 4.27)	0.602	0.892
19	81	2 (2.5%, 2/81)	0.582	0.975	0.63 (0.16, 2.55)	0.517	0.870
20	68	1 (1.5%, 1/68)	0.366	0.975	0.39 (0.05, 2.76)	0.343	0.798
21	58	3 (5.2%, 3/58)	0.740	0.975	1.07 (0.34, 3.37)	0.908	0.987
22	105	3 (2.9%, 3/105)	0.625	0.975	0.75 (0.24, 2.37)	0.627	0.892
23	46	2 (4.3%, 2/46)	1	1	1.02 (0.25, 4.14)	0.975	0.987
24	68	4 (5.9%, 4/68)	0.542	0.975	1.90 (0.70, 5.14)	0.208	0.749
25	51	2 (3.9%, 2/51)	1	1	1.01 (0.25, 4.10)	0.987	0.987
26	5	0	/	/	/	/	/
27	73	4 (5.5%, 4/73)	0.561	0.975	1.17 (0.43, 3.17)	0.756	0.893
28	81	2 (2.5%, 2/81)	0.582	0.975	0.62 (0.15, 2.52)	0.505	0.870
29	45	4 (8.9%, 4/45)	0.145	0.975	1.73 (0.64, 4.68)	0.283	0.749
30	87	2 (2.3%, 2/87)	0.585	0.975	0.44 (0.11, 1.79)	0.252	0.749
31	93	2 (2.2%, 2/93)	0.435	0.975	0.46 (0.11, 1.87)	0.279	0.749
32	7	1 (14.3%, 1/7)	0.289	0.975	0.69 (0.10, 4.97)	0.716	0.893
33	37	2 (5.4%, 2/37)	0.677	0.975	0.78 (0.19, 3.17)	0.733	0.893
34	236	10 (4.5%, 10/223)	1	1	0.98 (0.51, 1.88)	0.960	0.987
35	55	2 (3.6%, 2/55)	1	1	1.27 (0.31, 5.14)	0.738	0.893
36	66	3 (4.5%, 3/66)	0.764	0.975	1.56 (0.50, 4.92)	0.444	0.865
37	126	10 (7.9%, 10/126)	0.073	0.900	2.72 (1.42, 5.19)	0.003	0.031
38	107	9 (8.4%, 9/107)	0.057	0.900	3.26 (1.65, 6.44)	0.001	0.012
39	66	4 (6.1%, 4/66)	0.535	0.975	2.48 (0.92, 6.73)	0.074	0.545
40	81	1 (1.2%, 1/81)	0.260	0.975	0.52 (0.07, 3.71)	0.513	0.870
41	92	11 (12.0%, 11/92)	0.003	0.110	5.79 (3.11, 10.77)	< 0.001	< 0.001
42	34	2 (5.9%, 2/34)	0.661	0.975	0.35 (0.09, 1.41)	0.140	0.749
SUM	2690	117	/	/	/	/	/

^a^P1 value—Fisher′s test.

^b^P2 FDR‐adjusted values—*p* values were adjusted for multiple comparisons using the Benjamini–Hochberg false discovery rate (FDR) method.

^c^IRR—incidence rate ratio from Poisson′s regression.

^d^P3 value—Fisher′s test‐unadjusted *p* value derived from Poisson regression.

^e^P4 FDR‐adjusted values—*p* value adjusted for multiple comparisons using the Benjamini–Hochberg false discovery rate (FDR) method based on Poisson′s regression.

For the 23 novel variants identified in the SCMC cohort, nine were splicing variants, 10 were frameshift variants, and four were missense variants. Splicing variants and frameshift variants are truncating mutations and can lead to a nonfunctional protein. For missense variants, c.3610G>T and c.4709G>T of the *TSC2* gene have the same location but different amino acid changes with known pathogenic variants (c.3610G>A and c.4709G>C). Variants c.560T>C and c.775T>C of the *TSC1* gene have not been previously reported. They are rare variants that had no recording in the GnomAD and the 1000 Genomes databases. And they were estimated to have a deleterious effect on protein function by SIFT, PolyPhen‐2, and MutationTaster. Thirteen variants were identified with the CR phenotype in the SCMC cohort. Among them, eight were detected in the prenatal period, four were infants (< 3 years old), and one was a child. Five of them (38%, 5/13) had neurological comorbidities.

We summarized the phenotype of CR‐related variants (Supporting Information 2: Table S2). Almost one‐third (28.7%, 41/143) were identified in the fetus, and one‐third were identified in the infants (36.4%, 52/143). For those patients who only reported a CR phenotype, most are neonates or infants (72.7%, 24/33). These results indicated that CR presented early. No special characteristics of phenotype in Exon 15 variants or Exon 18 variants in *TSC1* and Exon 41 in *TSC2* were discovered.

## 4. Discussion

In this study, we demonstrated partial enrichment of CR‐related variants in specific exons, including Exon 15 and Exon 18 of the *TSC1* gene and Exon 41 in the *TSC2* gene. Exon 15 is the dominant exon containing a large number of the variants in *TSC1* [14]. The copy number variant within Exon 15 is also reported in a CR patient [15]. In *TSC1*, Exon 15 encodes the terminal part of the Rho‐activating domain (Amino Acids 145–510) [16]. Exon 15 is also the largest exon in *TSC1*. The Rho‐activating domain is the binding site to Rho proteins. The Rho family of small GTPases regulates the ensuing actin cytoskeletal organization at focal adhesions. Loss of function of this domain disrupts the cell matrix adhesion and may initiate the development of TSC hamartomas [17]. However, the mechanism to explain how Rho‐activating domain dysfunction causes the CR phenotype has not been well illustrated.

Exon 18 is located in the coiled‐coil region (Amino Acids 730–996). The coiled‐coil structure functions as an attractive protein folding motif for the fabrication of self‐assembled, responsive, and bioactive materials and is important for TSC1–TSC2 interaction and stabilization [18, 19]. Exon 18 is the essential exon in this domain [20]. Although the direct relationship between the coiled‐coil region and CR has not yet been reported, one study established a mouse model with Exons 17 and 18 loss in *Tsc1* in ventricular myocytes by MLC2vcreKI (Cre recombinase). They did not describe the rate of mice presenting CR, but they found larger left ventricular diameters by echocardiographic analysis in mutant mice. Pathology analyses found PAS and pS6 stains in heart tissue, indicating the CR. These results support the important role of Exon 18.

Nearly all exons of TSC2 have CR‐related variants. This may be due to the significant effects of TSC2 in heart development. On the other hand, the TSC1–TSC2 complex is reported as the critical inhibitor of mTORC1 and activator of mTORC2 [21]. The mTOR signaling pathway is involved in the heart with complex effects, including ischemia, metabolic cardiomyopathies, and cardiac remodeling [22]. This is the vital connection between TSC1/TSC2 and the heart. The TSC2 protein contains three domains, including the N‐terminal TSC1‐interacting region (Amino Acid Residues 55–469), the tuberin‐type domain (Amino Acid Residues 555–903), and the GTPase activator (GAP) domain (Amino Acid Residues 1562–1748) [23]. Exon 41 encodes the terminal part of the GAP domain. This domain works as the sole catalytically active domain in the TSC1–TSC2 complex [24]. So, a deficiency of TSC2 may have a severe effect on the mTOR signaling pathway. In both human beings and mouse models, P/LP variants of *TSC2* cause a more severe phenotype than *TSC1* [25]. Further investigations are necessary to validate these potential genotype–phenotype associations.

The TSC is a complex disease with various phenotypes. Different mutations in the same codon with different amino acid changes cause different phenotypes [26]. Even patients with the same variant in one family had different phenotypes [27]. It is hard to predict the symptoms based on genotype [28]. Only a few clues have been addressed. Both Au et al. and Kothare et al. reported more severe neurological symptoms in TSC2 patients than in TSC1 patients [29, 30]. Muto et al. report that patients with variants located in premature termination codons had a younger onset age and more severe phenotype than those with missense variants [31]. Variants in Exons 22 and 23 of TSC2 seem to cause a mild phenotype with a reduced risk of infantile spasm [32]. Variants in Exons 25–31 of TSC2 are less likely to cause classic TSC features [33]. This study hopes to provide clues that may help reduce the challenges encountered in genetic counseling [12].

In this study, we relied on ClinVar, HGMD, and LOVD databases. Due to reporting bias, variants associated with more severe phenotypes are more likely to be reported and curated, whereas variants with milder or absent phenotypes may be underrepresented. Therefore, the variants included in this study may not fully represent the true phenotypic spectrum. Accordingly, the associations identified here may reflect over‐representation rather than definitive genotype–phenotype relationships. This was the limitation of this study. On the other hand, we tried to summarize the CR phenotype of those variants. However, no unique characteristics of the CR phenotype in particular were identified. This may be due to a lack of detailed information about the location, size, and follow‐up information of CR. As a result, many detailed questions still have no answer, like the relationship between onset age, parental disease condition, and CR phenotypes with variants in other regions. TSC is relatively more prevalent compared with other rare disorders. Therefore, further validation using population‐based datasets or prospective cohorts is warranted to confirm these findings.

The genotype–phenotype observation in this study may help genetic counseling and alert physicians to pay attention: for those variants located in hotspots, physicians can suggest echocardiography or cardiac magnetic resonance imaging to determine the presence of CR. On the other hand, most neonatal or infantile TSC patients have solely the CR phenotype. With the development of prenatal ultrasound and MRI tests, more fetal CR has been identified. The diagnosis time point of TSC is earlier than before. Earlier evaluation of medical treatment can be assessed, and patients can benefit from it. Sudden death related to CR in the early postnatal period can be avoided. The third point is that, as the mechanism of CR has not been well established, our finding can provide clues for investigating the underlying mechanism and may contribute to the treatment.

## Author Contributions

X.C. and R.Y. wrote the main manuscript text. X.C. and J.L. prepared the figures. W.S., Y.C., L.F., and F.B. revised the manuscript. F.B. supervised this program. All authors reviewed the manuscript. X.C. and R.Y. are cofirst authors.

## Funding

No funding was received for this manuscript.

## Disclosure

All authors agree to give consent for the publication of this manuscript.

## Ethics Statement

This study has been approved by the ethics committee of Shanghai Children′s Medical Center (SCMCIRB‐K2020060‐1). Pretest counseling was performed by clinical physicians. Informed consent was obtained from the patient′s parents.

## Conflicts of Interest

The authors declare no conflicts of interest.

## Supporting Information

Additional supporting information can be found online in the Supporting Information section.

## Supporting information


**Supporting Information 1** Table S1: All variants analyzed (after curation) in this study. All variants included in the final analyses, including 1250 *TSC1* variants and 2690 *TSC2* variants.

**Supporting Information 2** Table S2: Reported phenotype of CR‐related variants. This table lists all CR‐related variants (this study) reported phenotype. The phenotype has been researched through a literature review.

## Data Availability

The information on variants and clinical data listed in this article and supporting information used to support the findings of this study are available from the corresponding author upon request. The data are not publicly available due to privacy or ethical restrictions.
